# Lupus skin disease

**DOI:** 10.1186/ar4621

**Published:** 2014-09-18

**Authors:** Victoria P Werth

**Affiliations:** 1 University of Pennsylvania, Philadelphia, PA, USA; 2 Philadelphia VAMC, Philadelphia, PA, USA

## Skin findings in lupus erythematosus

The skin findings in lupus erythematosus include lupus-specific and lupus-nonspecific categorizations. Lupus specific includes chronic cutaneous, subacute cutaneous, and acute cutaneous lupus. Lupus-specific findings include having a skin biopsy that shows LE-specific histology. The diagnosis of cutaneous lupus can be made regardless of whether the patient meets ACR or SLICC criteria for lupus. Lupus-nonspecific skin findings refer to lesions such as vasculitis or urticaria, where the findings are not histopathologically distinct for lupus and/or may be seen as a feature of other disease processes beyond lupus erythematosus. Chronic cutaneous lupus includes localized, generalized, and hypertrophic lupus, lupus panniculitis, and papulomucinous lupus. There is currently an ongoing international Delphi approach to unify the classification of cutaneous lupus, since the ongoing proliferation of how best to group the various presentations of cutaneous lupus is confusing.

## Skin and SLE criteria

Four of the SLE criteria are dermatologic, and the number of skin criteria contributes to there being many skin predominant lupus patients who meet criteria for SLE. Another approach to how best to classify SLE was recently published by the SLICC group. There is recognition of the variety of skin lesions that can be seen with the new criteria, but some criteria such as alopecia may be difficult in terms of attribution to lupus. The specificity of the new criteria will require ongoing investigation. In addition, some patients with cutaneous lupus initially do progress to SLE, but recent data suggest that, during progression to SLE, the SLE criteria are often met with skin, arthritis, hematologic, and serologic findings.

## Pathophysiology and triggers of cutaneous lupus erythematosus

The pathophysiologic findings of cutaneous lupus include interface dermatitis, with dendritic cells, CD4 and CD8 lymphocytes, and activation of innate immune proteins, including antimicrobial peptides. An interferon signature is seen in the skin and frequently in the blood with patients with cutaneous lupus, and this correlates with the activity of lupus in the skin. There is evidence that medications are a frequent trigger of subacute cutaneous lupus, with about one-third of patients having drugs as a trigger or aggravating factor. In particular, medications such as terbinafine, TNF inhibitors, and omeprazole, in addition to usual culprits such as thiazide, should be considered as risk factors.

## Quality of life and cutaneous lupus erythematosus

Recent studies indicate an extremely large impact of cutaneous lupus on quality of life, particularly related to activity of the skin disease. Studies with the SF-36 demonstrate that domains related to mental health, role emotion, and social function are worse in cutaneous lupus than in type II diabetes and recent myocardia infarction. Sixty percent of cutaneous lupus patients are depressed.

## Measurement of disease severity in cutaneous lupus erythematosus

The cutaneous lupus erythematosus area and severity index (CLASI) is a way to measure skin severity. The CLASI has undergone many validation studies for inter-rater and intra-rater reliability, responsiveness, correlation with QoL, and correlation with disease biomarkers. The CLASI has been studied in many different ethnic and racial groups, and has now been used in large international multicenter trials. These studies have demonstrated the importance of smoking as a risk factor for onset and severity in cutaneous lupus, as well as lack of responsiveness to current treatments.

## Treatment of cutaneous lupus erythematosus

There is still a paucity of successful trials for cutaneous lupus. Hydroxychloroquine works for 50 to 60% of patients. Addition of quinacrine to hydroxychloroquine can improve response in two-thirds of patients refractory to hydroxychloroquine alone. There is a correlation of hydroxychloroquine levels with response. Lenalidomide appears to have been beneficial in two small open-label trials of refractory cutaneous lupus. Rituximab has helped some patients with refractory bullous lupus, a disease normally mediated by antibodies against type VII collagen. With the improved understanding of pathogenesis, measures of disease severity, and the understanding of the impact of lupus skin disease on patients, there is increasing interest in improving the approaches to treatment for patients.

